# Potential Mechanisms Responsible for the Antinephrolithic Effects of an Aqueous Extract of Fructus Aurantii

**DOI:** 10.1155/2015/491409

**Published:** 2015-06-15

**Authors:** Xiaoran Li, Qiang Liang, Yunji Sun, Long Diao, Ze Qin, Wei Wang, Jianzhong Lu, Shengjun Fu, Baoliang Ma, Zhongjin Yue

**Affiliations:** ^1^Department of Urology, Institute of Urology, Gansu Nephro-Urological Clinical Center, Key Laboratory of Urological Diseases in Gansu Province, The Second Hospital of Lanzhou University, Lanzhou, Gansu 730030, China; ^2^Department of Neurosurgery, The Neurosurgery Clinical Medical Center, The Second Hospital of Lanzhou University, Lanzhou, Gansu 730030, China

## Abstract

The potential effects of Fa extract on the prevention and treatment of CaOx nephrolithiasis were analyzed in an ethylene glycol- (EG-) induced CaOx crystallization model in rats and an *in vitro* assay. Multiple biochemical variables were measured in the urine and kidney. Kidney sections were subjected to histopathological and immunohistochemical analyses. Urolithiasis-related osteopontin (OPN) was evaluated by Western blotting. The *in vitro* assay revealed the significant inhibition of crystal formation (3.50 ± 1.43) and dilution of formed crystals (12.20 ± 3.35) in the group treated with 1 mg/mL Fa extract compared with the control group (52.30 ± 4.71 and 53.00 ± 4.54, resp.) (*p* < 0.05). The *in vivo* experiments showed that prophylactic treatment with Fa aqueous extract significantly prevented EG-induced renal crystallization and pathological alterations compared with nephrolithic rats (*p* < 0.05). Significantly lower levels of oxidative stress, oxalate, and OPN expression as well as increased citrate and urine output levels were observed in both the low- and high-dose prophylactic groups (*p* < 0.05). However, in the low- and high-dose therapeutic groups, none of these indexes were significantly improved (*p* > 0.05) except for urinary oxalate in the high-dose therapeutic groups (*p* < 0.05). Fa extract prevented CaOx crystallization and promoted crystal dissolution *in vitro*. Additionally, it was efficacious in preventing the formation of CaOx nephrolithiasis in rats.

## 1. Introduction

Urolithiasis is a worldwide public health problem, and the economic burden of urinary calculi is enormous. The lifetime incidence of urolithiasis is 5–12%, and approximately 7% of women and 13% of men are affected by this condition [1]. Approximately 80% of kidney stones are composed of calcium oxalate. The mechanisms involved in stone formation are not fully understood. It is generally agreed that urolithiasis involves events such as crystal nucleation as well as aggregation and growth of insoluble particles [2]. Urine is supersaturated with common stone-forming minerals; however, the crystallization-inhibiting capacity of urine does not permit urolithiasis in most individuals. This natural inhibition capacity is deficient in stone-forming individuals [3]. Stone formation has a multifactorial etiopathogenesis involving anatomic, environmental, genetic, infectious, metabolic, nutritional, and socioeconomic factors that are major areas of concern [4].

The current treatments for urolithiasis, including the use of extracorporeal shock wave lithotripsy and surgical intervention for stone destruction, are widely applied in clinical practice to effectively manage urolithic patients. However, the 1-year recurrence rate in the majority of patients after these treatments is approximately 10%, and these procedures are invasive and do not prevent the recurrence of urolithiasis [5]. Certain drugs, such as citrate and thiazide diuretic preparations, may have side effects that compromise their long-term use and are not consistently effective.

Medicinal plants have played a significant role in various ancient traditional systems of medication. Even today, plants provide a cheap source of medicine for the majority of the world's population, and such medicines are considered quite safe, with minimal or no side effects [6]. Studies indicate that numerous herbs, including* Selaginella lepidophylla*,* Adiantum capillus veneris* Linn.,* Rubia cordifolia* roots, and* Cynodon dactylon* plants, can be used as antiurolithic agents [7–10]. Fructus Aurantii (Fa), the unripe fruit of* Citrus aurantium* Linn. (Rutaceae), is a Qi-regulating drug used in traditional Chinese medicine to treat urolithiasis clinically. Previous studies have shown that numerous bioactive compounds, including polymethoxylated flavones (PMFs), flavonoid glycosides, alkaloids, and coumarins, have been identified and isolated from Fructus Aurantii [11]. Studies have shown that certain components of Fructus Aurantii demonstrate pharmacological activity; antioxidant, antimicrobial [11], and anticarcinogenic properties [12, 13]; and neuroprotective effects [14, 15]. Fructus Aurantii has recently attracted the attention of many researchers, and the study of this herb is gradually increasing.

In the present study, we investigated the antiurolithiatic effects of an aqueous extract of Fructus Aurantii on calcium oxalate stones and its possible mechanisms of action using both* in vitro* and* in vivo* methods.

## 2. Materials and Methods

### 2.1. Plant Materials

Fructus Aurantii was purchased from the Second Hospital of Lanzhou University (Lanzhou, China) and graciously identified by members of the School of Pharmacy at Lanzhou University. A long-lasting decoction is used in traditional Chinese medicine, and the antioxidant levels from medicinal plants are not influenced by aqueous extraction; thus, the traditional decoction method was used to examine the effects of the aqueous extract [16]. Methods for extracting active ingredients from plants have been previously described [17]. Briefly, 500 g of raw material was mixed with 2.5 L of distilled water for 60 min and decocted for approximately 25 min. The supernatant was collected after filtration. Subsequently, 1.5 L of fresh distilled water was added to the residue, and the mixture was boiled. Finally, we combined the two aqueous extracts. After filtration, concentration, and lyophilization, the freeze-dried powder was stored at −80°C until use (yield = 23 g).

### 2.2. *In Vitro* Crystallization Assay

A previously described method was used to study the* in vitro* effects of the Fructus Aurantii aqueous extract on calcium oxalate crystals [18]. Briefly, stock solutions of 15 mL of calcium chloride (8.5 mM) and 15 mL of sodium oxalate (1.5 mM) containing 200 mM NaCl and 10 mM sodium acetate were adjusted to pH 5.7 to achieve calcium oxalate crystallization. A crystal formation inhibition test and crystal dissolution test were conducted. In the crystal formation inhibition test, solutions containing the Fructus Aurantii extract were added concomitantly to the solution containing the crystallization reagents at time 0 h (before crystallization) to obtain a final Fructus Aurantii concentration of 0.3, 0.7, or 1.0 mg/mL. The same amount of vehicle (distilled water) was added to solutions containing the crystallization reagents at time 0 h and was used for the control (0 mg/mL). All samples were maintained under 500 rpm agitation at 37°C for 24 h. For the dissolution test, crystal formation was induced over 24 h as previously described. After crystal formation, solutions of the Fructus Aurantii extract were added to obtain a final concentration of 0.3, 0.7, or 1.0 mg/mL. The same amount of vehicle (distilled water) was added to the formed crystals and was used for the control group (0 mg/mL). Each sample was observed under an inverted microscope (Nikon Corporation, Tokyo, Japan) to determine crystal morphology (400x). Five randomly selected fields were counted 24 h after the addition of the Fructus Aurantii extract or distilled water. The tests were performed as independent triplicates for each extract concentration.

### 2.3. Animals and Experimental Protocols

The study was approved by the Institutional Animal Care and Use Committee of Lanzhou University. Adult male Sprague-Dawley rats (200–220 g) from the Animal Experimental Center of Lanzhou University were maintained under standard laboratory conditions (temperature: 25 ± 2°C, humidity: 50–60%, and light regime: 12 h light-dark cycle). The animals had free access to tap water and a standard diet. After acclimatization for 1 week, the rats were divided into 6 groups of 10 animals each. The control group received only filtered water* ad libitum*. The EG group received 1% (V/V) EG in filtered water* ad libitum* for 28 days. The two preventive groups included an EG + PFa220 group, which received 1% (V/V) EG in filtered water* ad libitum* for 28 days along with Fructus Aurantii extract at a dose of 220 mg/kg BW per day, and an EG + PFa660 group, which received 1% (V/V) EG in filtered water* ad libitum* for 28 days along with Fructus Aurantii extract at a dose of 660 mg/kg BW per day. The two therapeutic groups included an EG + TFa220 group, which received 1% (V/V) EG in filtered water* ad libitum* for 28 days with a Fructus Aurantii extract at a dose of 220 mg/kg BW per day during days 15–28, and an EG + TFa660 group, which received 1% (V/V) EG in filtered water* ad libitum* for 28 days with a Fructus Aurantii extract at a dose of 660 mg/kg BW per day during days 15–28.

All extracts and standards were given orally once per day.

### 2.4. Collection and Analysis of Urine

On day 28 of the experimental period, the rats were placed in separate metabolic cages to collect 24 h urine samples, and 0.04% sodium azide was added to the urine to prevent bacterial growth. Following volume and pH determinations, urine samples were centrifuged at 2,000 ×g for 5 min to remove debris, and the supernatants were stored at −80°C for the subsequent determination of calcium, magnesium, uric acid, oxalate, and citrate using commercially available kits (Biovision Ltd., Milpitas, CA, USA; Instruchemie Ltd., Netherlands). Urinary 8-IP (a product of lipid peroxidation) was measured using an enzyme-linked immunosorbent assay kit (Quantikine Assay, R&D Systems, USA) following the manufacturer's instructions.

### 2.5. Kidney Homogenate Analysis

A 0.1 g sample of renal tissue was mixed with 9 volumes of cold phosphate buffer (pH 7.4) and then homogenized. The homogenates were centrifuged at 16,000 ×g for 5 min at 4°C. The supernatants were measured for superoxide dismutase (SOD) and malondialdehyde (MDA) using two kits (Jiancheng Bioengineering Ltd., Nanjing, China).

### 2.6. Kidney Crystal Deposits and Pathological Examination

Histological studies were performed using a Nikon Eclipse E400 light microscope. Crystal deposits in the kidney were evaluated as previously described [19] using the following point values: no deposits = 0 points; crystal deposits in the papillary tip = 1 point; crystal deposits in the corticomedullary junction = 2 points; and crystal deposits in the cortex = 3 points. If crystal deposits were observed at multiple sites, the points were combined to provide a total score for each pathological section. The pathological alterations were semiquantified based on the area of injury, from 0 to 3, as follows: 0 = invisible lesions; 1 = tubule interstitial inflammatory infiltration with lesion area <20% and mild dilation of the tubules; 2 = tubule interstitial inflammatory infiltration with lesion area <40% and obvious dilation of the tubules; and 3 = tubule interstitial inflammatory infiltration with lesion area >40% and severe dilation of the tubules [17].

### 2.7. Microbiological Studies

At the time of sacrifice, microbiological studies were carried out on urine aspirated from the bladder. The kidneys were aseptically removed and transected. One-half of each kidney was homogenized separately in 5 mL of sterile normal saline solution (TRI Instruments). Dilutions (10^−1^, 10^−3^, and 10^−5^) of the tissue homogenates were cultured on plates, and the bacteria were enumerated after correction for the dilution factor. Standard microbiological techniques were used. The presence of more than 10^5^ colony-forming units per mL (cfu/mL) of urine indicated the presence of a urinary tract infection. Pyelonephritis was defined as the presence of ≥10^5^ cfu/g of kidney tissue [20].

### 2.8. Immunohistochemical Staining

After preservation, the kidney sections were boiled for antigen retrieval and treated with 3% H_2_O_2_ to remove endogenous peroxidases. After rinsing with PBS, goat serum was used to block the sections. The sections were incubated at 4°C overnight with a polyclonal OPN antibody (rabbit polyclonal to OPN, ab8448, Abcam, Cambridge, UK) (5 *μ*g/mL). After rinsing with PBS, the sections were incubated at 37°C with a polymer helper for 20 min. After rinsing three times with PBS, the slides were treated with poly-peroxidase-anti-rabbit/mouse IgG for 2 h before a final wash with PBS and staining with DAB.

### 2.9. Western Blot Analysis for OPN Protein in the Kidney

Kidney tissue (100 mg) was homogenized in 1 mL of lysis buffer mixed with 10 *μ*L of phenylmethanesulfonyl fluoride (100 mM) before being centrifuged (12,000 ×g 5 min) at 4°C. The supernatants were collected to determine the protein concentration. The proteins were mixed with SDS-PAGE sample loading buffer. The mixture was heated at 100°C for 5 min before performing SDS-PAGE with a 10% acrylamide resolving gel. The separated proteins were transferred to polyvinylidene fluoride (PVDF) membranes. The PVDF membranes were blocked with 5% skim milk powder for 2 h at room temperature before being incubated with OPN antibodies at 4°C overnight. The PVDF membranes were washed three times with TBST for 10 min each rinse before being incubated with antibodies labeled with horseradish peroxidase for 2 h. The membranes were then washed as previously described, and the proteins were detected by enhanced chemiluminescence.

### 2.10. Statistical Analyses

All of the data are expressed as the mean ± standard deviation except where otherwise is indicated. Significant differences were determined by Student's *t*-test for comparisons between two groups or one-way ANOVA for comparisons of three or more groups. The rates of urinary tract infection or pyelonephritis were compared between groups by *χ* ^2^ analysis. A *p* value of <0.05 was considered statistically significant.

## 3. Results

### 3.1. *In Vitro* Assay

**Figure 1:**
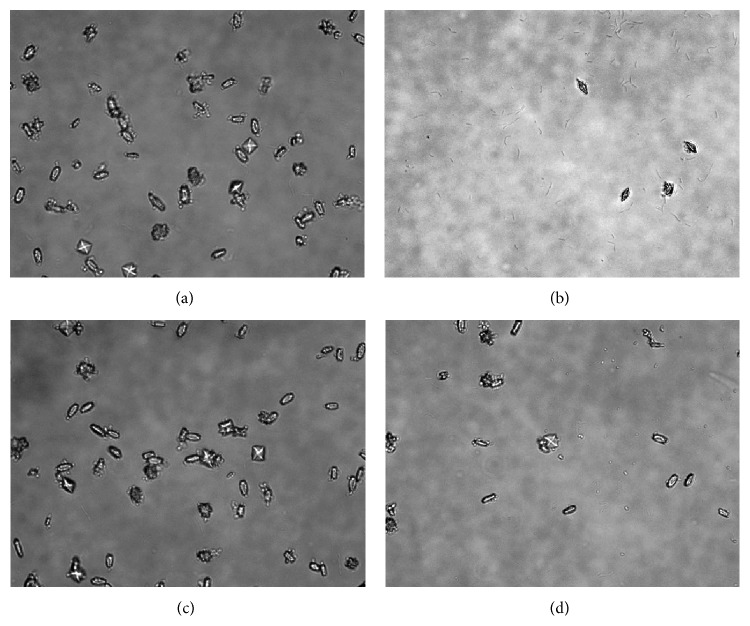
The CaOx crystals were observed under an inverted microscope (400x). (a) A control group exhibits crystal formation during the inhibition test. (b) The Fa extract was concomitantly added at a concentration of 1.0 mg/mL to the solution containing the crystallization reagents at time 0 h for a duration of 24 h. (c) A control group exhibits crystal formation during the crystal dissolution test. (d) The Fa extract was added at a concentration of 1.0 mg/mL to the formed crystals for 24 h.

**Figure 2:**
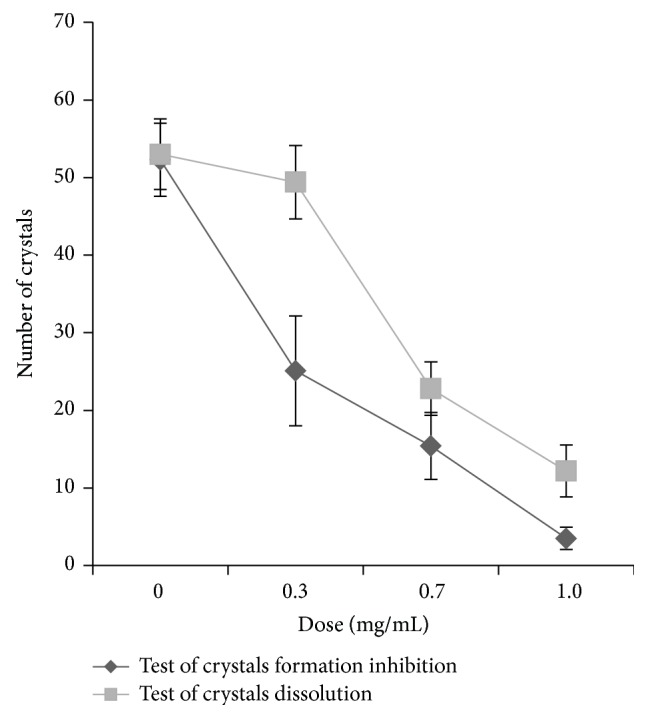
Number of crystals in crystal formation inhibition and crystal dissolution tests.

The crystal formation inhibition test showed numerous crystals composed predominately of claviform calcium oxalate in the control group (0 mg/mL) (Figure 1(a)). Crystal formation was significantly inhibited 24 h after the addition of the Fa extract at a concentration of 1.0 mg/mL to the solution containing the crystallization reagents at time 0 h (Figure 1(b)). The Fa extract caused a more prismatic shape in the calcium oxalate crystals (Figure 1(b)). The crystal counts obtained using a light microscope (400x) were 25.10 ± 7.09 (*p* < 0.05), 15.40 ± 4.32, and 3.50 ± 1.43 (*p* < 0.05) for solutions incubated with Fa at 0.3, 0.7, or 1.0 mg/mL, respectively, compared to the control (52.30 ± 4.71) (Figure 2). The crystal dissolution test revealed that the number and morphology of crystals from the control group were similar to those of crystals from the control group based on the crystal formation inhibition test (Figure 1(c)). After the addition of the Fa extract at 1.0 mg/mL to formed crystals for 24 h, the number of crystals was significantly reduced (Figure 1(d)). The results were significant at doses of 0.7 (22.80 ± 3.42) and 1.0 mg/mL (12.20 ± 3.35) when compared with the control group (control: 53.00 ± 4.54) (Figure 2).

### 3.2. Urinary Biochemical Variables

As shown in Table 1, the EG group had significantly higher urinary oxalate when compared with the control group. In addition, the urine volume was increased, and citrate levels were decreased. Preventive treatment with the Fa extract significantly decreased urinary oxalate and significantly increased urine output and citrate levels when compared with the EG group (*p* < 0.05). However, in the EG + TFa220 and EG + TFa660 groups, except for the significantly lower oxalate levels in the EG + TFa660 group, the above-mentioned variables did not improve significantly compared to those in the EG group.

### 3.3. Histology

**Figure 3:**
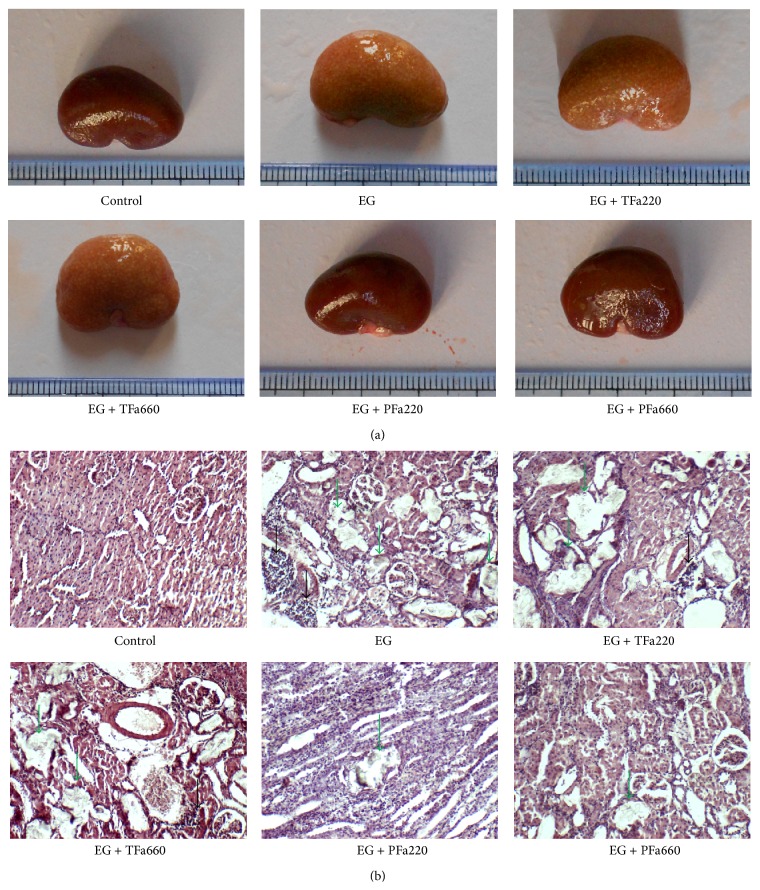
Kidney crystal deposits and pathological examination. (a) Gross anatomy of the kidney. (b) Micrograph of renal tissue (100x magnification).

**Figure 4:**
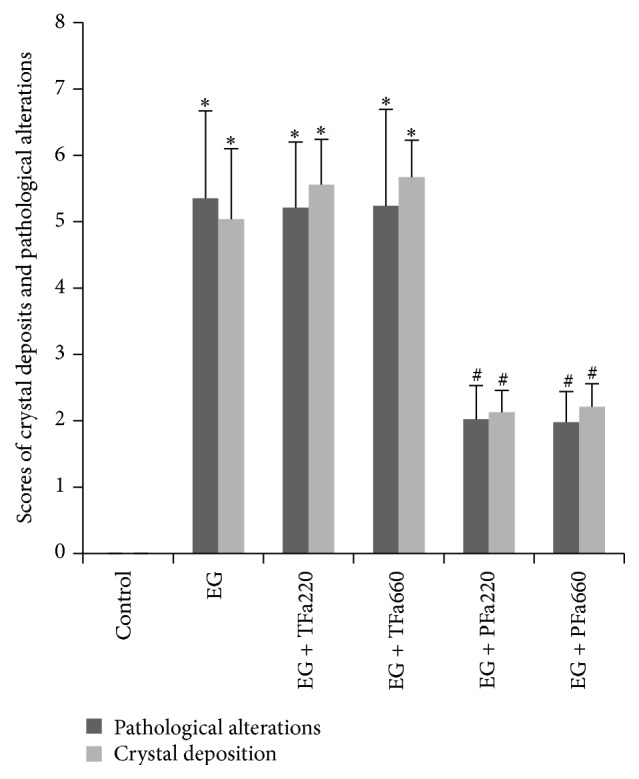
Scores for crystal deposition and pathological alterations. The columns and bars represent the mean ± SD (^*∗*^ *p* < 0.05 compared with the control, ^#^ *p* < 0.05 compared with the EG group, and ^@^ *p* < 0.05 for the EG + PFa660 group compared with the EG + PFa220 group; *n* = 10).

As shown in Figure 3(a), severe swelling, focal hemorrhage, and pale yellow needle-point crystals were observed in the kidneys of the EG group. Gross alterations similar to those observed in the EG group were observed in the EG + TFa220 and EG + TFa660 groups. However, only mild swelling and a small number of yellow needle-point crystals were observed in the EG + PFa220 and EG + PFa660 groups (Figure 3(a)). Severe dilation of the tubules and massive inflammatory infiltration (black arrows) were observed in the EG group (Figure 3(b)). The pathological alterations observed in the EG + PFa220 and EG + PFa660 groups included a slight dilation of the tubules and mild inflammatory infiltration (Figure 3(b)), and the pathological scores of the two groups were reduced compared with the EG group (Figure 4) (*p* < 0.05). However, no significant differences were observed between the EG + PFa220 and EG + PFa660 groups (Figure 4) (*p* > 0.05). The pathological scores of the EG + TFa220 and EG + TFa660 groups were significantly elevated compared with that of the control group (*p* < 0.05) (Figure 4).

### 3.4. Scoring of Kidney Crystal Deposits

Abundant crystals were observed in all regions of the kidney in the EG group, particularly in the cortex region, using a Nikon Eclipse E400 light microscope (green arrows) (Figure 3(b)). Compared with the EG group, significantly lower scores were not obtained for the EG + TFa220 group or the EG + TFa660 group (Figure 4) (*p* > 0.05). The number of crystal deposits was decreased in groups EG + PFa220 and EG + PFa660 compared with the EG group (Figure 4) (*p* < 0.05). However, no significant differences were observed between the EG + PFa220 and EG + PFa660 groups (Figure 4) (*p* > 0.05).

### 3.5. Microbiological Studies

None of the rats from the control group (0%) suffered from a urinary tract infection, whereas the rate of urinary tract infection in rats in the EG group was 100% (10 of 10). After preventive treatment with Fa extracts, the rates of urinary tract infection in rats in the EG + PFa220 and EG + PFa660 groups were 20% (2 of 10) and 10% (1 of 10), respectively (*p* < 0.05 compared with EG group). However, after therapeutic treatment with Fa extracts, the rates of urinary tract infection in rats in the EG + TFa220 and EG + TFa660 groups were 80% (8 of 10) and 90% (9 of 10), respectively (*p* > 0.05 compared with the EG group). The pyelonephritis rates in the control, EG, EG + PFa220, EG + PFa660, EG + TFa220, and EG + TFa660 groups (0%, 100%, 20%, 10%, 80%, and 90%, resp.) were consistent with the rate of urinary tract infection in each group.

### 3.6. Immunohistochemistry

**Figure 5:**
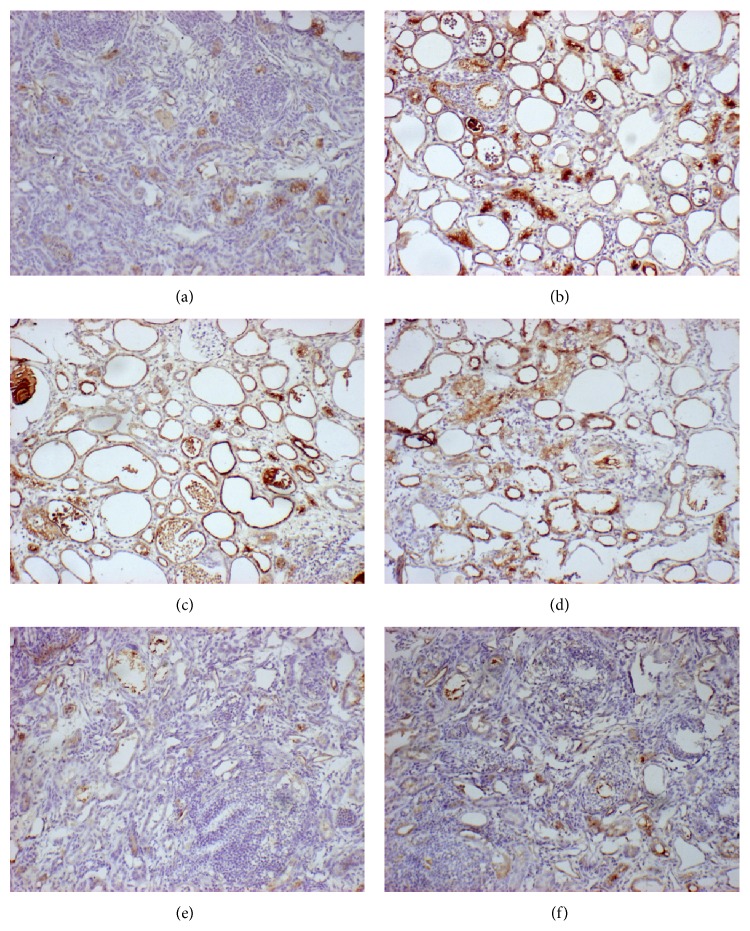
Immunostaining of OPN for each group (Figures 5(a)–5(f)) in kidney tissue (100x magnification).

Immunohistochemical staining for osteopontin revealed OPN expression in all groups (Figure 5); however, OPN levels were barely detectable in the control group (Figure 5(a)). The EG group (Figure 5(b)) exhibited OPN expression that was distributed throughout the renal tubular cells of the whole kidney, particularly in the distended tubules. The increased OPN synthesis in the renal tubules of the EG + TFa220 and EG + TFa660 groups (Figures 5(c) and 5(d), resp.) was similar to that of the EG group. However, the EG + PFa220 and EG + PFa660 groups (Figures 5(e) and 5(f), resp.) demonstrated mild OPN staining that was barely detectable.

### 3.7. Western Blot

**Figure 6:**
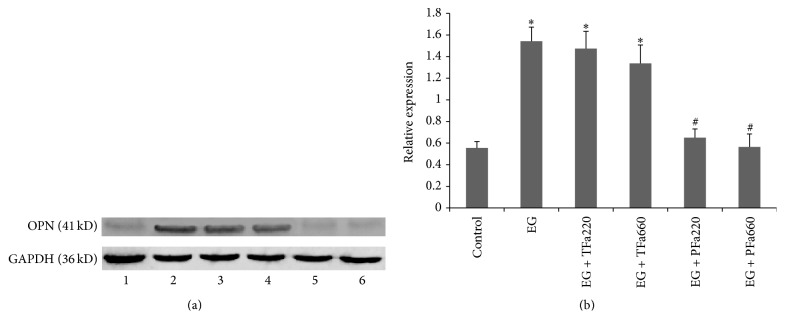
Western blots for OPN expression. (a) Analysis of OPN protein expression in the SD rat kidney was conducted using conventional western blotting. GAPDH was used as the internal control. The following groups are shown: (1) control group, (2) EG group, (3) EG + TFa220 group, (4) EG + TFa660 group, (5) EG + PFa220 group, and (6) EG + PFa660 group. (b) Quantitative densitometric analysis of OPN protein levels. The values are reported as the mean ± SD of three experiments (^*∗*^ *p* < 0.05 when compared with the control, ^#^ *p* < 0.05 when compared with the EG group, and ^@^ *p* < 0.05 for the EG + PFa660 group compared with the EG + PFa220 group).

Western blot analysis of OPN revealed distinct bands at 41 kDa in the EG group and the therapeutic groups (EG + TFa220 and EG + TFa660). No clear bands were observed in the control group or the prophylactic groups (EG + PFa220 and EG + PFa660) (Figure 6(a)). The corresponding band intensities of GAPDH are presented in Figure 6(a). The results of quantitative analysis of OPN expression by densitometry are expressed as the ratio of the OPN band intensity relative to the intensity of GAPDH. These data indicated that the EG group exhibited relatively high levels of OPN expression (Figure 6(b)). The therapeutically treated rat tissues (EG + TFa220 and EG + TFa660) exhibited OPN expression levels that were similar to those of the EG group. In contrast, the control and prophylactic groups (EG + PFa220 and EG + PFa660) exhibited minimal OPN expression (Figure 6(b)).

### 3.8. Oxidative Studies

As shown in Table 2, the EG group exhibited increased renal MDA and urinary 8-IP content and decreased SOD levels when compared with control animals (*p* < 0.05). Preventive treatment with the Fa extract protected against the oxidative stress induced by the lithogenic treatment (*p* < 0.05). The above three indicators were nearly restored to normal levels in the EG + PFa660 group. However, there were no statistically significant alterations in MDA, SOD, or 8-IP levels in any of the therapeutic groups when compared with the EG group.

## 4. Discussion

Based on its medicinal use, we evaluated the antiurolithic potential of a Fructus Aurantii extract using different models.

An* in vitro* crystal formation inhibition test showed that Fa decreased the crystal count and modified the morphology of CaOx crystals. Similar alterations in calcium oxalate crystal morphology have been reported with Mg^2+^ and citrate [21]. In the crystal dissolution study, Fa decomposed formed CaOx crystals in a concentration-dependent manner. These data are similar to those observed for potassium citrate, a well-known inhibitor of CaOx crystallization that is widely used for the clinical management of urolithiasis [3]. The crystal dissolution study suggests that the potential activity of Fa as a kidney stone inhibitor is associated with complexation of nonionized Ca^2+^ in CaOx crystal embryos, which ultimately leads to the dissolution of CaOx. Indeed, we are not aware of any study in which certain components of Fa aqueous extract have been reported to dissolve CaOx. However, it must be recognized that, under* in vitro* conditions, Fa may dissolve crystal embryos that might grow into stable nuclei, ultimately forming solid bonds. Although crystal production is not always equivalent to stone formation, crystal formation along the urinary tract is the primary requisite for subsequent stone development. Crystal retention has been identified as a key step in symptomatic stone formation. Thus, interference with crystal formation and crystal dissolution are important preventive and therapeutic strategies for kidney stones.

The EG-induced model has been widely used by researchers to study urinary calculi in rats [22–24]. Hepatic enzymes metabolize EG to oxalic acid, which combines with calcium ions in the renal tubule epithelium. Several biochemical abnormalities, including increased urine output, hyperoxaluria, hypomagnesiuria, hyperuricosuria, hypercalciuria, and hypocitraturia, have been observed in the urine of lithic animals [25].

In the present study, we assessed the preventive effects of an orally administered Fa aqueous extract on CaOx kidney stones in EG-treated rats. The Fa aqueous extract was administered to rats approximately halfway through kidney stone development to observe its potential therapeutic effects. The oral doses were selected based on the clinical dosage provided by the Chinese Pharmacopoeia and the ratio of body to surface area between the human and the rat. After oral treatment with the Fa aqueous extract, an improvement in urinary biochemistry, a decrease in OPN expression, and fewer renal depositions were observed compared with the EG-induced rats from the preventive group. There are multiple mechanisms of action that could produce these effects.

Citrate decreases calcium oxalate and calcium phosphate stone supersaturation via the formation of soluble calcium citrate [26]. Hypocitraturia is a common metabolic abnormality in nephrolithic patients [27]. In the present study, urinary citrate was decreased in EG-treated rats. Administration of the Fa extract enhanced citrate excretion and reduced crystal deposition. Therefore, hypercitraturic activity might be a potential mechanism involved in the antilithiatic action of Fa. Continuing hypercalciuria promotes the nucleation and subsequent precipitation of calcium oxalate crystals from the urine. Some studies have suggested that disorders of renal tubular calcium reabsorption are the major cause of hypercalciuria. In the present study, hypercalciuria was not observed in the EG group as previously described. A possible explanation for this result is that the formation of CaOx consumes free calcium (Ca) and normalizes Ca levels in the urine. Most importantly, the oxalate values were approximately normal in the EG + PFa660 group. Oxalate is a more important risk factor in the process of urinary calculi formation than hypercalciuria [28]. The normalization of oxalate excretion levels in the prophylactic groups suggests that the Fa extract exerts a protective effect against stone production. Additionally, the approximate 2-fold increase in urinary volume in the EG + PFa660 group compared with the EG group revealed distinct diuresis, which effectively diluted the oxalate, increased urine flow, and prevented CaOx retention.

In addition, crystals can induce the production of reactive oxygen species (ROS) in kidney tissue. The production of ROS can lead to renal epithelial injury, which increases the areas available for crystal attachment and eventual retention within the kidney [29, 30]. Antioxidant therapy has been used to manage CaOx nephrolithiasis [31, 32]. In previous studies, Selvam reported that vitamin E administration prevented crystal precipitation in the rat kidney [33]. Vitamin E has also been shown to decrease the urinary excretion of oxalate and calcium and restored antioxidant ability in the blood in patients who underwent surgical nephrolithiasis removal [34]. A previous study of antioxidant enzyme levels in lithic rats indicated that almost all the antioxidant enzyme activity was attenuated [35]. In our study, SOD was decreased and MDA and 8-IP were increased in EG-treated rats when compared with untreated rats (*p* < 0.05). After preventive treatment with Fa, SOD activity was restored, and MDA and 8-IP levels declined when compared with those in EG-treated rats (*p* < 0.05). Particularly in the EG + PFa660 group, the OS marker levels were approximately normal, demonstrating an optimal antioxidative effect of Fa at the chosen dose. This effect may be due to the flavonoids in the extract, which protect the kidney against the damaging effects of ROS, including peroxynitrite, peroxyl radicals, superoxide, singlet oxygen, and hydroxyl radicals [36].

Based on chemical compositional analysis and bacteriology of metabolic stones, studies have suggested that some metabolic stones, particularly CaOx nephrolithiasis, originate from bacterial infections [37–39]. Among these studies, Sohshang et al. reported that approximately 47 calculi obtained from 100 kidney stone patients had positive bacterial cultures in stone samples and urine; in addition,* Escherichia coli* was the most common bacteria causing infection [38]. Chutipongtanate et al. [40] demonstrated that CaOx crystal growth and aggregation can be promoted directly by bacteria* in vitro* and that CaOx stone promotion by bacteria is comparable to fragmented red blood cell membranes, which were recently proposed to promote CaOx stones [41]. Coumarins, bioactive compounds isolated from Fructus Aurantii, demonstrate exceptional antimicrobial activity. In the present study, the inhibition of CaOx crystal growth and aggregation by Fa extract may be due to its antimicrobial activity. In addition, in the* in vivo* experiments, all of the rats in the EG group (100%) suffered from urinary tract infections, which could have contributed to crystal nucleation [42]. Preventive use of Fa extract containing bioactive compounds that show exceptional antimicrobial activity can significantly reduce urinary tract infection, which may inhibit crystal formation. However, therapeutic treatment with Fa extracts did not significantly improve urinary tract infection, likely because the dose of drug we used did not reach therapeutic levels.

OPN is an important protein component of the stone matrix and is thought to be involved in many pathologic and physiologic processes, including cell migration, adhesion, inflammation, and renal injury [43–45]. OPN is localized in specific sites in the kidney and is mainly restricted to renal epithelial cells in the cortex. However, elevated OPN expression in the renal cortex and medulla was observed after CaOx crystal deposition [25, 45]. Immobilized OPN increases crystal aggregation, and OPN adherence to the surface of collagen granules causes an increase in calcium oxalate crystal adherence and aggregation [46]. Thus, factors that modulate renal OPN expression are expected to regulate the growth and aggregation of crystals in the kidney. In our study, we determined that preventive treatment with Fa extract significantly lowered OPN expression compared with EG-treated groups.

In the therapeutic groups, certain urolithiasis-related indicators were improved. However, the number of crystals was not significantly reduced. It is well known that the etiology of CaOx urolithiasis is complex and involves multiple factors. The improvement of certain indicators may not have been sufficient to reduce crystals that were already formed by day 14 in the EG + TFa220 and EG + TFa660 groups. Thus, renal crystal deposition did not significantly improve in the EG + TFa220 and EG + TFa660 groups. However, further studies are needed to confirm this hypothesis.

## 5. Conclusion

In conclusion, our results indicate that Fa aqueous extract prevents CaOx crystallization and promotes crystal dissolution. The extract also prevents CaOx calculi in the rat kidney, possibly through a combination of increasing antioxidant levels and antimicrobial activity while decreasing urinary stone-forming constituents, OPN expression, and hypercitraturic effects. Further studies are necessary to identify the active components in the extract and the mechanisms responsible for the observed pharmacological activity.

## Figures and Tables

**Table 1:** Urine biochemical variables ($\overset{-}{x}\pm s$).

Parameter (unit)	Control	EG	EG + TFa220	EG + TFa660	EG + PFa220	EG + PFa660
pH	6.40 ± 0.40	6.57 ± 0.49	6.53 ± 0.28	6.40 ± 0.57	6.49 ± 0.36	6.45 ± 0.25
Urine volume (mL)	8.58 ± 1.03	10.38 ± 2.21	9.33 ± 1.43	12.31 ± 2.37^*∗*^	18.66 ± 2.86^*∗*#^	20.36 ± 2.80^*∗*#^
Oxalate (mmol/L)	0.71 ± 0.14	2.24 ± 0.44^*∗*^	1.92 ± 0.48^*∗*^	1.08 ± 0.25^#^	1.00 ± 0.19^#^	0.82 ± 0.17^#^
Calcium (mmol/L)	2.29 ± 0.23	2.22 ± 0.61	1.95 ± 0.54	2.08 ± 0.61	2.12 ± 0.5	2.28 ± 0.29
Citrate (mmol/L)	0.73 ± 0.21	0.64 ± 0.14	0.70 ± 0.15	0.92 ± 0.22	1.46 ± 0.36^*∗*#^	1.78 ± 0.44^*∗*#^
Uric acid (mmol/L)	1.19 ± 0.24	1.36 ± 0.35	1.25 ± 0.22	1.28 ± 0.16	1.11 ± 0.25	1.10 ± 0.28
Magnesium (*μ*mol/L)	4.61 ± 0.67	5.01 ± 0.59	5.15 ± 0.60	5.11 ± 0.53	5.39 ± 0.69	4.80 ± 0.55

Values are expressed as the mean ± SD. ^*∗*^ *p* < 0.05 when compared with the control, ^#^ *p* < 0.05 when compared with the EG group, and ^@^ *p* < 0.05 for the EG + PFa660 group compared with the EG + PFa220 group. P: preventive; T: therapeutic.

**Table 2:** Oxidative stress variables in urolithiasis rats treated with Fa extract ($\overset{-}{x}\pm s$).

Parameter (unit)	Control	EG	EG + TFa220	EG + TFa660	EG + PFa220	EG + PFa660
8-IP (pg/mL)	3.03 ± 0.60	9.34 ± 1.77^*∗*^	8.70 ± 2.16^*∗*^	9.58 ± 2.51^*∗*^	3.64 ± 1.19^#^	2.59 ± 1.07^#^
SOD (U/mg)	302.87 ± 65.57	148.16 ± 31.84^*∗*^	162.13 ± 45.17^*∗*^	145.78 ± 45.80^*∗*^	284.99 ± 66.02^#^	311.69 ± 25.67^#^
MDA (nmol/mg)	1.07 ± 0.30	3.17 ± 0.45^*∗*^	3.33 ± 0.62^*∗*^	3.14 ± 0.88^*∗*^	0.98 ± 0.24^#^	1.09 ± 0.18^#^

Values are expressed as the mean ± SD. ^*∗*^ *p* < 0.05 when compared with the control, ^#^ *p* < 0.05 when compared with the EG group, and ^@^ *p* < 0.05 for the EG + PFa660 group compared with the EG + PFa220 group. P: preventive; T: therapeutic.

## Abbreviations

Fa: Fructus Aurantii
OS: Oxidative stress
SD: Sprague-Dawley
SOD: Superoxide dismutase
ROS: Reactive oxygen species
MDA: Malondialdehyde
MAPK: Mitogen-activated protein kinase
ERK: Extracellular signal-regulated kinase
OPN: Osteopontin
BUN: Blood urea nitrogen
Cr: Serum creatinine
JNK: c-Jun NH_2_-terminal protein kinase
CaOx: Calcium oxalate
EG: Ethylene glycol.

## References

[1] Preminger G. M., Tiselius H.-G., Assimos D. G. (2007). 2007 guideline for the management of ureteral calculi. *European Urology*.

[2] Baumann J. M. (1998). Stone prevention: why so little progress?. *Urological Research*.

[3] Tiselius H.-G. (2003). Epidemiology and medical management of stone disease. *BJU International*.

[4] Rathod N. R., Biswas D., Chitme H. R., Ratna S., Muchandi I. S., Chandra R. (2012). Anti-urolithiatic effects of Punica granatum in male rats. *Journal of Ethnopharmacology*.

[5] Pak C. Y. C. (1998). Kidney stones. *The Lancet*.

[6] Bashir S., Gilani A. H. (2009). Antiurolithic effect of Bergenia ligulata rhizome: an explanation of the underlying mechanisms. *Journal of Ethnopharmacology*.

[7] Mirian E.-C. M., Juanita N.-M., Christophe B. O., Estela M.-C. M. (2013). Molecular mechanisms involved in the protective effect of the chloroform extract of *Selaginella lepidophylla* (Hook. et Grev.) Spring in a lithiasic rat model. *Urological Research*.

[8] Ahmed A., Wadud A., Jahan N., Bilal A., Hajera S. (2013). Efficacy of *Adiantum capillus veneris* Linn in chemically induced urolithiasis in rats. *Journal of Ethnopharmacology*.

[9] Divakar K., Pawar A. T., Chandrasekhar S. B., Dighe S. B., Divakar G. (2010). Protective effect of the hydro-alcoholic extract of *Rubia cordifolia* roots against ethylene glycol induced urolithiasis in rats. *Food and Chemical Toxicology*.

[10] Atmani F., Sadki C., Aziz M., Mimouni M., Hacht B. (2009). Cynodon dactylon extract as a preventive and curative agent in experimentally induced nephrolithiasis. *Urological Research*.

[11] Chen H.-F., Zhang W.-G., Yuan J.-B., Li Y.-G., Yang S.-L., Yang W.-L. (2012). Simultaneous quantification of polymethoxylated flavones and coumarins in *Fructus aurantii* and *Fructus aurantii* immaturus using HPLC-ESI-MS/MS. *Journal of Pharmaceutical and Biomedical Analysis*.

[12] Li S., Pan M.-H., Lai C.-S., Lo C.-Y., Dushenkov S., Ho C.-T. (2007). Isolation and syntheses of polymethoxyflavones and hydroxylated polymethoxyflavones as inhibitors of HL-60 cell lines. *Bioorganic & Medicinal Chemistry*.

[13] Manthey J. A., Guthrie N. (2002). Antiproliferative activities of citrus flavonoids against six human cancer cell lines. *Journal of Agricultural and Food Chemistry*.

[14] Akao Y., itoh T., Ohguchi K., Iinuma M., Nozawa Y. (2008). Interactive effects of polymethoxy flavones from Citrus on cell growth inhibition in human neuroblastoma SH-SY5Y cells. *Bioorganic and Medicinal Chemistry*.

[15] Nakajima A., Yamakuni T., Haraguchi M. (2007). Nobiletin, a citrus flavonoid that improves memory impairment, rescues bulbectomy-induced cholinergic neurodegeneration in mice. *Journal of Pharmacological Sciences*.

[16] Li H.-B., Jiang Y., Wong C.-C., Cheng K.-W., Chen F. (2007). Evaluation of two methods for the extraction of antioxidants from medicinal plants. *Analytical and Bioanalytical Chemistry*.

[17] Mi J., Duan J., Zhang J., Lu J., Wang H., Wang Z. (2012). Evaluation of antiurolithic effect and the possible mechanisms of *Desmodium styracifolium* and *Pyrrosiae* petiolosa in rats. *Urological Research*.

[18] Kulaksızoğlu S., Sofikerim M., Çevik C. (2008). In vitro effect of lemon and orange juices on calcium oxalate crystallization. *International Urology and Nephrology*.

[19] Yamaguchi S., Wiessner J. H., Hasegawa A. T., Hung L. Y., Mandel G. S., Mandel N. S. (2005). Study of a rat model for calcium oxalate crystal formation without severe renal damage in selected conditions. *International Journal of Urology*.

[20] Barros M., Martinelli R., Rocha H. (2008). Experimental supratrigonal cystectomy: II—evaluation of urinary calculi, infection, and bladder dysfunction in the pathogenesis of renal failure. *International Urology and Nephrology*.

[21] Guerra A., Meschi T., Allegri F. (2006). Concentrated urine and diluted urine: the effects of citrate and magnesium on the crystallization of calcium oxalate induced in vitro by an oxalate load. *Urological Research*.

[22] Itoh Y., Yasui T., Okada A., Tozawa K., Hayashi Y., Kohri K. (2005). Preventive effects of green tea on renal stone formation and the role of oxidative stress in nephrolithiasis. *Journal of Urology*.

[23] Aydin H., Yencilek F., Mutlu N., Çomunoğlu N., Koyuncu H. H., Sarica K. (2010). Ethylene glycol induced hyperoxaluria increases plasma and renal tissue asymmetrical dimethylarginine in rats: a new pathogenetic link in hyperoxaluria induced disorders. *The Journal of Urology*.

[24] Alex M., Sauganth Paul M. V., Abhilash M., Mathews V. V., Anilkumar T. V., Nair R. H. (2014). Astaxanthin modulates osteopontin and transforming growth factor *β*1 expression levels in a rat model of nephrolithiasis: a comparison with citrate administration. *BJU International*.

[25] Khan S. R. (1997). Animal models of kidney stone formation: an analysis. *World Journal of Urology*.

[26] Chow K., Dixon J., Gilpin S., Kavanagh J. P., Rao P. N. (2004). Citrate inhibits growth of residual fragments in an in vitro model of calcium oxalate renal stones. *Kidney International*.

[27] Hamm L. L., Hering-Smith K. S. (2002). Pathophysiology of hypocitraturic nephrolithiasis. *Endocrinology and Metabolism Clinics of North America*.

[28] Borghi L., Meschi T., Amato F., Briganti A., Novarini A., Giannini A. (1996). Urinary volume, water and recurrences in idiopathic calcium nephrolithiasis: a 5-year randomized prospective study. *Journal of Urology*.

[29] Khan S. R. (2011). Crystal/cell interaction and nephrolithiasis. *Archivio Italiano di Urologia, Andrologia*.

[30] Khan S. R. (2013). Reactive oxygen species as the molecular modulators of calcium oxalate kidney stone formation: Evidence from clinical and experimental investigations. *Journal of Urology*.

[31] Lee H.-J., Jeong S.-J., Park M. N. (2012). Gallotannin suppresses calcium oxalate crystal binding and oxalate-induced oxidative stress in renal epithelial cells. *Biological and Pharmaceutical Bulletin*.

[32] Kim H. B., Shanu A., Wood S. (2011). Phenolic antioxidants tert-butyl-bisphenol and vitamin e decrease oxidative stress and enhance vascular function in an animal model of rhabdomyolysis yet do not improve acute renal dysfunction. *Free Radical Research*.

[33] Selvam R. (2002). Calcium oxalate stone disease: role of lipid peroxidation and antioxidants. *Urological Research*.

[34] Sumitra K., Pragasam V., Sakthivel R., Kalaiselvi P., Varalakshmi P. (2005). Beneficial effect of vitamin E supplementation on the biochemical and kinetic properties of Tamm-Horsfall glycoprotein in hypertensive and hyperoxaluric patients. *Nephrology Dialysis Transplantation*.

[35] Huang H. S., Ma M. C., Chen J., Chen C. F. (2002). Changes in the oxidant-antioxidant balance in the kidney of rats with nephrolithiasis induced by ethylene glycol. *Journal of Urology*.

[36] Pietta P.-G. (2000). Flavonoids as antioxidants. *Journal of Natural Products*.

[37] Takeuchi H., Okada Y., Yoshida O., Arai Y., Tomoyoshi T. (1989). Urinary tract infection associated with urinary calculi. 1. The significance of urinary tract infection in urinary calculi. *Acta Urologica Japonica*.

[38] Sohshang H. L., Singh M. A., Singh N. G. B., Singh S. R. (2000). Biochemical and bacteriological study of urinary calculi. *The Journal of Communicable Diseases*.

[39] Tavichakorntrakool R., Prasongwattana V., Sungkeeree S. (2012). Extensive characterizations of bacteria isolated from catheterized urine and stone matrices in patients with nephrolithiasis. *Nephrology Dialysis Transplantation*.

[40] Chutipongtanate S., Sutthimethakorn S., Chiangjong W., Thongboonkerd V. (2013). Bacteria can promote calcium oxalate crystal growth and aggregation. *Journal of Biological Inorganic Chemistry*.

[41] Chutipongtanate S., Thongboonkerd V. (2010). Red blood cell membrane fragments but not intact red blood cells promote calcium oxalate monohydrate crystal growth and aggregation. *Journal of Urology*.

[42] Kajander E. O., Ciftcioglu N., Miller-Hjelle M. A., Hjelle J. T. (2001). Nanobacteria: controversial pathogens in nephrolithiasis and polycystic kidney disease. *Current Opinion in Nephrology and Hypertension*.

[43] Xie Y., Sakatsume M., Nishi S., Narita I., Arakawa M., Gejyo F. (2001). Expression, roles, receptors, and regulation of osteopontin in the kidney. *Kidney International*.

[44] Hudkins K. L., Giachelli C. M., Cui Y., Couser W. G., Johnson R. J., Alpers C. E. (1999). Osteopontin expression in fetal and mature human kidney. *Journal of the American Society of Nephrology*.

[45] Khan S. R., Johnson J. M., Peck A. B., Cornelius J. G., Glenton P. A. (2002). Expression of osteopontin in rat kidneys: induction during ethylene glycol induced calcium oxalate nephrolithiasis. *Journal of Urology*.

[46] Konya E., Umekawa T., Iguchi M., Kurita T. (2003). The role of osteopontin on calcium oxalate crystal formation. *European Urology*.

